# Mercurial Inflammation of the Gums, and Treatment

**Published:** 1873-03

**Authors:** N. E. Hollace

**Affiliations:** Boston, Mass.


### ARTICLE III.

### *Mercurial Inflammation of the Gums, and Treatment.*

BY N. E. HOLLACE, M. D., BOSTON, MASS.

The frequently repeated introduction of mercury into the system, when taken in sufficient quantity, gives rise to the development of peculiar morbid phenomena in the gums and other parts of the mouth. The first indication of the specific action of this powerful medicinal agent upon the animal economy consists in a slightly increased redness and tumefaction of the free edges of the gums around the necks of the inferior incisors, while the investing mucous membrane of the adherent portion, a little lower down, often assumes a white color, owing to the opacity of the epithelium.

These appearances are soon followed by increased secretion of saliva, a brassy or coppery taste, soreness of the teeth and gums, inflammation and swelling of the mucous membrane of the mouth, fauces, cheeks, and salivary glands, and a peculiarly offensive odor of breath.

In the meantime the edges of the gums about the necks of the teeth swell and assume an increase of redness; the saliva becomes viscid, and is secreted in such abundance as to flow from the mouth, and the movements of the jaws are attended with much pain.

The alveolo-dental periosteum is thickened, and the teeth raised from their sockets and loosened. A vesicular eruption sometimes appears, followed by ulceration and sloughing of the gums, and very frequently by necrosis of large portions of the alveolar processes and jaw bone.

I have frequently had occasion to remove portions both of the superior and inferior maxillary bones, the necrosis having been occasioned by the use of this medicine. By the prudent administration of mercury, however, salivation may be induced without causing the deplorable effects just described. But the specific action of this agent upon the constitution is always attended by more or less tumefaction and sponginess of the gums; and when once brought under its influence, however perfectly its effects may have subsided, they are ever after more susceptible to morbid impressions.

Treatment.—It is scarcely necessary to say that until the use of this mineral is discontinued, it will be impossible to remove, or even counteract its effects upon the gums. But in mild cases thse symptoms soon disappear after the action which it has produced on the general system has completely subsided. But when the gums continue spongy the bowels should be kept open with saline cathartics, the patient restricted to a plain diet, and the mouth gargled several times a day with demulcent decoctions and mild astringent lotions, to which it may sometimes be advisable to add a little laudanum. Washes made from chloride of soda or lime may be used to correct the excessive fetor of the breath. After the action of the medicine upon the system has subsided, and the disease assumed a chronic form, the gums should be freely scarified by passing a lancet entirely through their substance between the teeth, and this operation should be repeated as often as every four or five days, until they are completely restored.

The use of astringent washes should at the same time be continued, and if there are any teeth which, from the loss of their vitality, or from having become very much loosened by the partial destruction of their sockets, act as irritants, they should be removed. When the gums have ulcerated, the application of a strong solution of sulphate of zinc, or nitrate of silver, applied with a camel's hair pencil, will prove beneficial.

Permanganate of potassa, applied to the mucous membrane, if in an ulcerated condition, is one of the best disinfectants; as a gargle it may be used in the strength of from one to four scruples to a pint of water.

---

